# Corneal epithelial cell biocompatibility to silicone hydrogel and conventional hydrogel contact lens packaging solutions

**Published:** 2010-02-19

**Authors:** M.B. Gorbet, N.C. Tanti, L. Jones, H. Sheardown

**Affiliations:** 1 Systems Design Engineering, University of Waterloo, Waterloo, Ontario, Canada; 2 Centre for Contact Lens Research, School of Optometry, University of Waterloo, Waterloo, Ontario, Canada; 3 Department of Chemical Engineering, McMaster University, Hamilton, Ontario, Canada

## Abstract

**Purpose:**

Although all contact lenses (CLs) are applied initially to the eye directly from a packaging solution, little is known about the effects of these solutions on human corneal epithelial cells (HCECs). Due to the porous nature of CL materials, they have the potential to sorb components of the packaging solution during storage, which could then be subsequently released upon insertion of the CL on the eye. The purpose of this study was to investigate the effect of various packaging solutions on HCECs, using an in vitro model.

**Methods:**

An in vitro assay was developed whereby various silicone hydrogels and conventional, poly-2-hydroxyethylmethacrylate  (polyHEMA)-based lens materials were removed directly from their packaging and then incubated for up to 24 h with HCECs. The effect of the retained and released packaging solution components on HCECs was assessed by measuring cell viability, adhesion phenotype, and apoptosis.

**Results:**

Incubation of HCECs with CLs stored in borate-buffered packaging solutions resulted in a significant reduction in cell viability. Adherent cells incubated with these CLs also exhibited reduced levels of β_1_ and α_3_ integrin. Soaking borate-buffered packaged CLs in PBS before cell incubation resolved viability and integrin expression in all cases, with the exception of galyfilcon A and balafilcon A, from which a 20% reduction in cell viability was still observed. In comparison, CLs stored in phosphate-buffered packaging solutions had cellular viability and expression of integrins similar to control cells (cells incubated in the absence of a lens). When incubated with cells at a 10% concentration in serum-free medium, borate-buffered packaging solutions and borate-containing saline (Unisol 4) significantly reduced cell viability and integrin expression. Neither caspase activation nor annexin V binding was observed on cells following exposure to borate buffer solution. However, a significant decrease in reactive oxygen species was observed at 24 h. These latter results suggest that in vitro exposure to low concentration of borate/boric acid results in cell dysfunction, leading to necrosis rather than apoptosis.

**Conclusions:**

Borate-buffered packaging solutions were shown to adversely affect the viability and integrin expression of HCECs in vitro. When used in ophthalmic packaging solutions, the antimicrobial properties of borate buffer may be outweighed by its relatively cytotoxic effects on cells.

## Introduction

Chemical properties such as oxygen permeability [1] and wettability [2], in addition to protein and lipid sorption [3-6], have been the primary focus of most studies investigating the biocompatibility of contact lens materials with the external ocular surface. Recently, potential issues with various components of multi-purpose cleaning solutions and the preservative agents contained therein have led to in vitro studies whereby these solutions, at various concentrations, are tested directly on conjunctival or epithelial cells [7-11]. Contact lens material parameters such as water content, the presence of various functional groups, surface treatment, and the nature of the underlying polymeric matrix can affect the uptake and subsequent release of various components from care regimens that come into contact with the lens materials [12]. A recent study with benzalkonium chloride, a common preservative used in ophthalmic solutions, demonstrated the in vitro cytoxicity of extracts from contact lenses soaked in benzalkonium chloride solutions [8]. A significant effect of the lens material on the release of cytotoxic components was found, which further suggests that interactions between contact lens materials and the solutions that they contact may have deleterious effects on the cornea.

Although all contact lenses are initially applied to the eye directly from the packaging container in which the lens is supplied, literature available on the direct effect of packaging solutions on the ocular surface remains sparse. Due to the highly porous nature of hydrogel materials, soft contact lenses have the potential to take up significant quantities of the components of ophthalmic solutions [12-14], which can be subsequently released upon insertion onto the ocular surface. The effects of these components on corneal epithelial cells have not been widely studied. One study reported the effect of borate versus phosphate buffered packaging solutions on lens parameters [15]. While phosphate and borate buffers have been used extensively in ophthalmic solutions, there is limited knowledge of their biological effect on corneal epithelial cells. Borate salts have been reported to have both cytotoxic and anti-inflammatory effects on cells, depending on the borate salt, its concentration, and the type of cells used [16-18]. A recent study also reported that corneal epithelial cells treated with 1% boric acid displayed discontinued tight junctions in vitro [9]. The potential cytotoxic effects of borate buffers on corneal epithelial cells is a specific concern for users of disposable lenses, since they are removed from their packaging solution and inserted onto the ocular surface daily. Commercially available conventional, polyHEMA-based hydrogel and silicone hydrogel contact lenses are stored in either phosphate or borate buffered packaging solutions.  Therefore, this in vitro study was undertaken to investigate the effect of lens release on corneal epithelial cells. The specific cellular effects studied were corneal epithelial cell adhesion phenotype and viability.

## Methods

### Reagents and antibodies

The keratinocyte, serum-free medium and supplement were from ScienCell (Carlsbad, CA). All other cell culture reagents, Dulbecco’s minimum essential medium, fetal bovine serum, phosphate buffer saline, and TriplExpress were purchased from Invitrogen (Burlington, Canada). Monoclonal antibodies to β_1_ integrin (CD29; Immunotech-Coulter, Marseilles, France) and β_4_ integrin (CD104; Serotec, Mississauga, Canada) and ICAM-1 (CD54; Immunotech-Coulter) were fluorescein isothiocyanate (FITC) conjugates. Monoclonal antibodies against Fas (CD95; Immunotech-Coulter), α_3_ integrin (CD49c; Serotec) and α_6_ integrin (CD49f; Serotec) were R-phycoerythrin conjugates. To determine if, following incubation with the contact lenses, cells were undergoing apoptosis, caspase activation was measured by flow cytometry using FITC-VAD-FMK (EMD Biosciences, San Diego, CA). Annexin V-FITC and YO-PRO-1 were from Molecular Probes (Eugene, OR), 2’-7’ dichlorohydrofluorescein diacetate (DCF-A) from Biotium (Hayward, CA), and Rhodamine 123 from Invitrogen. Paraformaldehyde was purchased from Fisher Scientific (Ottawa, Ontario, Canada) and all other chemicals used to prepare Hepes Tyrode Buffer were of analytical or reagent grade (Sigma-Aldrich, Oakville, Canada). A sterile solution of Unisol 4® (unpreserved borate-buffered saline; Alcon, Fort Worth, TX) was purchased from a commercial source and used within its expiration date.

### Contact lenses

Both silicone hydrogel (SH) and polyHEMA-based conventional hydrogel (CH) lenses were tested. Table 1 lists the disclosed information on the lenses and packaging solutions used in the study. All lenses were obtained in their original packaging from the manufacturer and had 14.0–14.2 mm diameters and 8.50–8.70 mm base curvatures. Unworn lenses were used in one of three states: directly from the packaging container (no treatment), after being rinsed 3× in sterile unpreserved PBS (PBS-rinsed), and after being soaked in sterile PBS for 24 h at room temperature (PBS-soaked). All lens treatments were performed under sterile conditions.

**Table 1:** Lens characteristics and disclosed packaging solution contents.

**Manufacturer**	**Brand name**	**Lens material (USAN)**	**Water content (%)**	**Buffering agent**	**Disclosed packaging additive**
Bausch & Lomb	*PureVision*	*balafilcon A*	*36*	*Borate*	
	SofLens 38	polymalcon	38	Phosphate	0.1% polyvinyl alcohol
	SofLens 66	alphafilcon A	66	Borate	
CIBA Vision	*Night & Day*	*lotrafilcon A*	*24*	*Phosphate*	
	Focus Dailies	nelfilcon A	69	Phosphate	0.02% poloxamer
	*O_2_Optix*	*lotrafilcon B*	*33*	*Phosphate*	*0.02% poloxamer*
Johnson & Johnson	1-Day Acuvue (etafilcon A-1day)	etafilcon A	58	Borate	
	*Acuvue Advance*	*galyfilcon A*	*47*	*Borate*	*≤0.01% methyl cellulose*
	*Acuvue OASYS*	*senofilcon A*	*38*	*Borate*	*≤0.01% methyl cellulose*

Silicone hydrogel lenses are italicized.

### In vitro cell culture

#### Immortalized human corneal epithelial cells

SV40-immortalized human corneal epithelial cells (HCECs) were a gift from Dr. M. Griffith (Ottawa Eye Research Institute, Ottawa, Canada) and have been characterized as human corneal epithelial cells previously [19]. Cells were cultured in keratinocyte serum-free medium supplemented with bovine pituitary extract, recombinant epidermal growth factor, and pen-strep (KSFM). Fresh medium was added every other day, and cells were grown to 90% confluency in tissue culture-treated flasks. Adherent cells were removed using the dissociation solution TriplExpress (Invitrogen).

#### In vitro model

An in vitro model, similar to that reported by Maltseva et al. [20], was developed. HCECs were seeded onto a 24-well tissue culture-treated polystyrene plate. Cells were left to adhere for 3 h in a humid CO_2_ incubator [21]. Supernatant was then removed and fresh serum-free medium was added. Sterile CH and SH lenses were placed gently on top of the monolayer, with the concave surface facing upwards and incubated for up to 24 h at 37 °C (5% CO_2_ in a humid incubator). Lenses were totally immersed in medium in all cases. After 24 h, the lenses were carefully removed from the wells. The lenses did not adhere to the HCEC monolayer, and HCECs did not proliferate on the contact lenses. In some cases, instead of testing a contact lens, up to 70 μl of sterile packaging solution or Unisol 4 was directly added to the adherent cells (corresponding to maximum final concentration of 10% in medium),followed by 8–24 h incubation.

Control experiments included experiments in which cells were also left to adhere for 24 or 48 h before incubation with contact lenses or borate solution, to determine if initial experimental conditions had a significant effect on the observed cytotoxic results. The effect of serum (10% final concentration) on cytotoxicity was also determined.

### Cellular viability

To evaluate the cytotoxicity of the products released by the contact lenses, the 3-(4,5-Dimethylthiazol-2-yl)-2,5-diphenyltetrazolium bromide (MTT) assay cellular viability assay was performed [8]. After a gentle rinse in sterile PBS, cells were incubated with a solution of 3-(4,5-dimethylthiazol-2-yl)-2,5-diphenyltetrazolium bromide (MTT at 1mg/ml in KSFM medium). After 3 h at 37 °C, cells were lysed with dimethyl sulfoxide (DMSO) and absorbance read at 595 nm [22,23]. All results are expressed as relative viability compared to cells grown in the absence of a contact lens.

### Cellular activation and apoptosis

To determine cellular activation and change in integrin expression induced by the presence of the contact lens and release of packaging solution components, HCECs were removed from the wells with TriplExpress (Invitrogen). Cells were washed and resuspended in Dulbecco's Modified Eagle Medium/fetal bovine serum. Small aliquots (25 μl) of HCECs, diluted in DMEM-FBS, were incubated with saturating concentration of fluorescently labeled antibodies for 1 h at 4 °C. Samples were then diluted in Hepes Tyrode Buffer (137 mM NaCl, 2.7 mM KCl, 16 mM NaHCO_3_, 5 mM MgCl_2_, 3.5 mM Hepes, 1g/l glucose, 2 g/l bovine serum albumin, pH 7.4), fixed in 1% paraformaldehyde, and analyzed by flow cytometry within 5 days.

To investigate the potential mechanisms involved in the cytotoxicity, several markers of apoptosis were studied. For caspase activation, 1 µl of FITC-VAD-FMK (Calbiochem, San Diego, CA) was added to samples, and they were incubated at 37 °C with 5% CO_2_. Following incubation and proper washing, samples were analyzed immediately on the flow cytometer [24]. Binding of annexin V-FITC on the cell membrane and permeability to YO-Pro-1 were also investigated. Annexin V binds to phosphatidylserine, which becomes exposed to the outer leaflet of the apoptotic cell membranes. YO-PRO-1 is a fluorescent DNA probe that is permeable through the membrane of apoptotic cells. Permeability to YO-PRO-1 has been linked to pores formed following apoptosis induced by activation of the P2X7 cell death receptor [25] or reactive oxygen species production [26]. Cells were incubated with 1 μl of each fluorescent dye in separate tubes for 30 min, washed, and analyzed immediately on the flow cytometer. To further characterize the potential mechanisms involved in cell death, the intracellular levels of reactive oxygen species were also measured using DCF-A. DCF-A was dissolved in DMSO at 4 mg/ml and further diluted in KSFM to 0.08 mg/ml. A small aliquot of cells (30 μl) were stained with DCF-A (final concentration 4 μg/ml) at 37 °C for 30 min. In parallel, as a positive control, cells were also stained with DCF-A in the presence of Phorbol 12-Myristate 13-Acetate (a potent protein kinase C [PKC] activator) to further stimulate reactive oxygen species production. Loss of mitochondria membrane potential was assessed using Rhodamine 123 (Rh123). A stock solution of Rh123 was diluted in ethanol to 1 mg/ml and further diluted in KSFM to 10 μg/ml on the day of the experiments. Rh123 was added to cells (final concentration 2 μM) and incubated at 37 °C for 30 min. Both Rh123 and DCF-HA were analyzed immediately by flow cytometry following the 37 °C incubation.

All flow cytometric measurements were acquired on a Becton Dickinson FACSVantage flow cytometer (Mountain View, CA) using CELLQuest Software (Becton Dickinson, Mountain View, CA). Appropriate isotype controls were used with each experiment. Data analysis was performed using FACSExpress (DeNovo Software, Los Angeles, CA).

### Statistical analysis

All results are reported as means±standard deviation (SD). To evaluate the significance of the differences in cell viability and cell activation, analysis of variance (ANOVA) was performed, followed by a post hoc Tukey test using the statistical analysis software Statistica (Tulsa, OK). A p value of less than 0.05 was required for statistical significance. The number of experiments was equal to or greater than three.

## Results

### Cell viability

**Figure 1:**
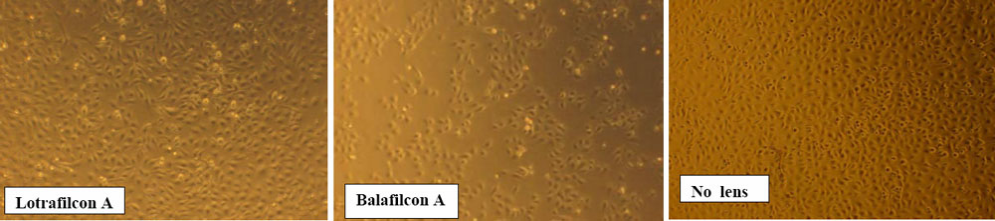
Micrographs of adherent human corneal epithelial cells after 24 h incubation with a contact lens. Corneal epithelial cells were incubated for 24 h in the presence of lotrafilcon A, directly out of its phosphate-buffered packaging solution and balafilcon A, directly out of its borate-buffered packaging solution. No lens represents the control cells, i.e., corneal epithelial cells grown in the absence of a contact lens.

Following 24 h incubation with lenses stored in borate-buffered packaging solutions, microscopic evaluation revealed the appearance of bare patches within the cell monolayer, suggesting cell death and/or impaired adhesion (Figure 1). In comparison, no difference could be observed between HCECs exposed to lotrafilcon A lenses and cells cultured in the absence of a lens.

After 24 h incubation with contact lens materials directly out of the packaging solution, a 40% reduction or more in cell viability (p<0.001) was observed for all contact lenses stored in borate-buffered packaging solutions (Table 2). In comparison, incubation in the presence of lenses stored in phosphate-buffered packaging solution did not lead to significantly reduced cell viability. These results were further confirmed with HCECs that were left to adhere for up to 48 h before incubation with the daily disposable lenses nelfilcon A (phosphate-buffered solution) and etafilcon A-1 day (borate-buffered solution; Table 3). Furthermore, the presence of serum did not have a protective effect against cytotoxicity that was observed with etafilcon A-1 day (Table 3).

**Table 2:** Viability of HCEC following a 24 h incubation with contact lenses and the effect of release from contact lenses stored in borate or phosphate buffered packaging solutions.

**Lens storage conditions**	**Lens**	**Viability**
Lens stored in borate buffered packaging solution	balafilcon A	56±7*
	alphafilcon A	59±7*
	etafilcon 1 day	63±10*
	galyfilcon A	55±8*
	senofilcon A	60±8*
Lens stored in phosphate buffered packaging solution	nelfilcon A	100±6
	lotrafilcon A	88±7
	lotrafilcon B	90±8
	polymalcon	98±3

Viability was measured by MTT assay and is expressed as a percentage relative to cells incubated for the same time in the absence of a contact lens (control). n=3 to 5, the asterisk indicates significantly different from cells grown in the absence of a lens p<0.01.

**Table 3:** Effect of experimental conditions on cell viability following incubation with a contact lens or test solution.

**Test sample**	**3 h settling**	**3 h settling + 10% FBS**	**24 h settling**	**48 h settling**
Etafilcon A	63±10*	60±11*	73±3*	71±4*
Nelfilcon A	94±5	98±8	92±10	100±4
Unisol (10%)	60±4*	ND	68±5*	74±5*#
PBS (10%)	102±6	ND	101±6	98±7

After the mentioned settling time, cells were incubated for 24 h with the test material or solution. Viability was measured by MTT assay at the end of the incubation period and expressed relative to control (cells grown in the absence of lens and solution). The asterisk indicates significantly different from lotrafilcon A (lens stored in a phosphate buffered solution), p<0.003. The sharp indicates significantly different from incubation with Unisol after a 3 h settling period p=0.032. ND indicates not determined.

**Figure 2:**
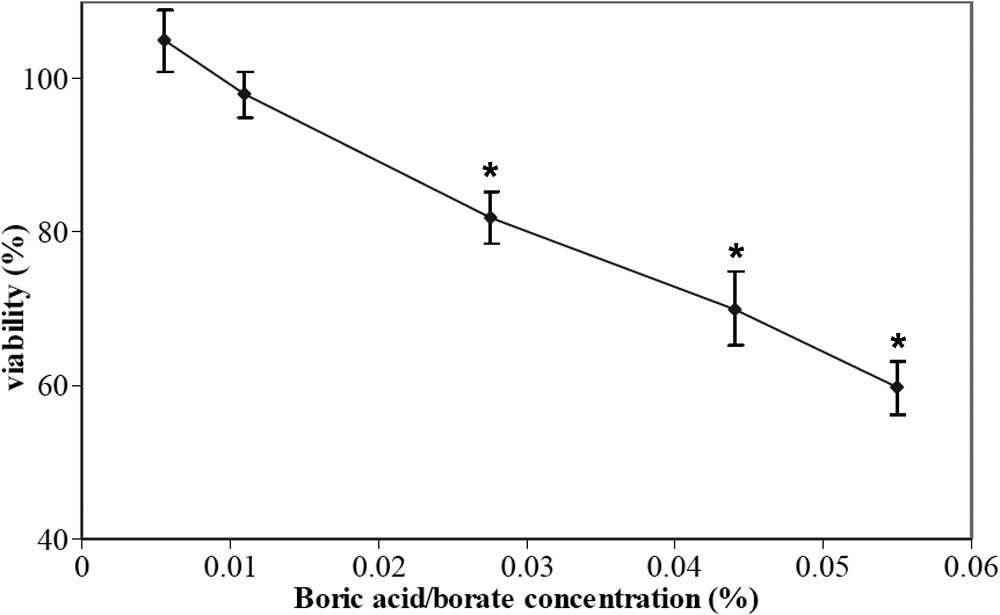
Effect of borate/boric acid on HCEC viability. Cells were incubated for 24 h with different concentrations of Unisol, a saline solution containing borate/boric acid. Viability was measured by 3-(4,5-Dimethylthiazol-2-yl)-2,5-diphenyltetrazolium bromide (MTT) assay and is expressed as a percentage relative to cells grown in the absence of a solution (control); n=3–4. The asterisk indicates significant difference from control, p<0.001.

The cytotoxicity to HCECs of borate-buffered packaging solutions was then directly assessed by adding sterile packaging solutions from galyfilcon A and balafilcon A lenses to the culture medium (10% final concentration). As illustrated in Table 4, a 10% dilution of solutions containing borate led to a significant reduction in cell viability after both 8 and 24 h exposures. Interestingly, at 8 h, a similar reduction in cell viability was observed between 10% borate buffer solution and products released from a contact lens. Yet at 24 h, a significantly more pronounced reduction in cell viability was observed with the direct addition of the packaging solution to the medium, compared to the change in viability observed with release from the etafilcon A-1day and balafilcon A lenses. Other packaging solutions from lenses listed in Table 1 were also tested on HCECs (data not shown), which confirmed the results presented in Table 4. Adding phosphate-buffered packaging solution (from comfilcon A, nelfilcon A, polymacon, and lotrafilcon B) to the medium (10% final concentration) did not affect cell viability (p>0.05). As packaging solutions contain additives and preservative agents that may affect the observed cytotoxicity, Unisol 4 (Alcon), an unpreserved borate-buffered solution, was tested at various concentrations on cells. The solution contained 0.5% boric acid and 0.052% sodium borate, as well as 0.66% sodium chloride (H. Ketelson, Alcon, personal communication, 2009). The overall concentration of borate/boric content of Unisol was 0.55%, for the results presented herein. When compared to exposure to PBS and no solution, a significant effect from borate buffer on cell viability was observed at all concentrations above 0.025% (p<0.03; Figure 2). The effect of the concentration was consistent over the different culture conditions (3 h, 24 h, and 48 h of culture prior to testing; data not shown).

**Table 4:** Effect of packaging solution on corneal epithelial cell viability as measured by the MTT assay.

**Contact time**	**Packaging solution from**			
	**etafilcon 1 day (borate based)**	**balafilcon A (borate based)**	**lotrafilcon A (phosphate based)**	**Borate buffer solution (Unisol 4)**
8 h	70±6*	76±1*	100	ND
24 h	45±2*	43±2*	91±4	60±4*

All solutions were diluted to a final concentration of 10% in KSFM. Results are expressed as a percentage compared to the control (cells incubated in KSFM medium only). The asterisk indicates significantly different from cells grown in the absence of a lens p≤0.04; ND indicates not determined. Note; incubation with etafilcon 1 day lens resulted in a viability of 75±7* at 8 h and 60±6* at 24 h.

**Figure 3:**
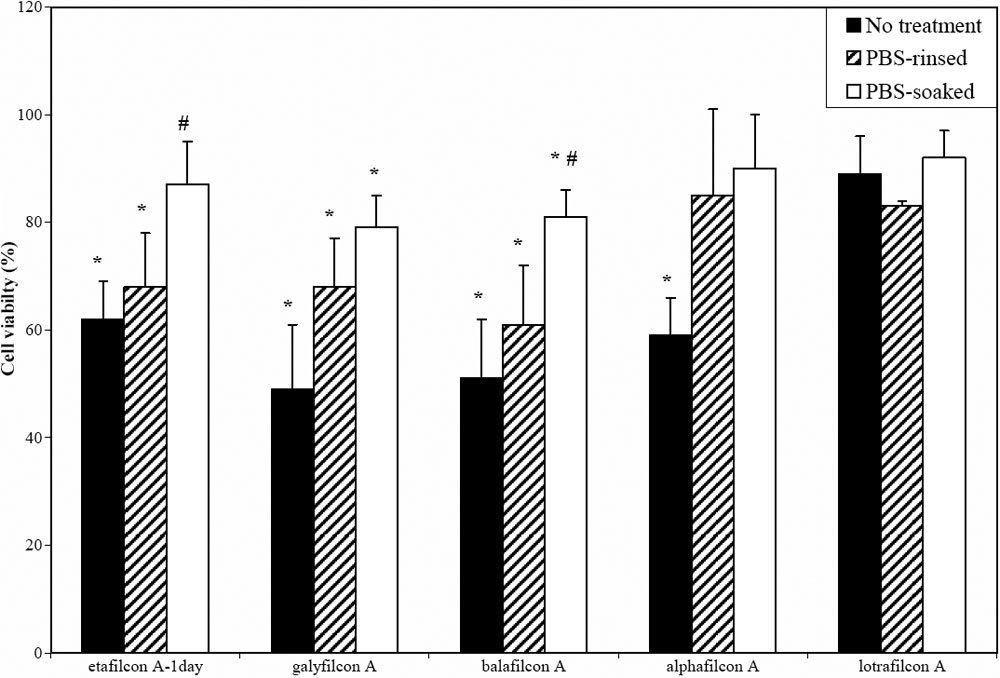
Effect of PBS rinse or soak on viability of corneal epithelial cells exposed to contact lenses. Prior to the cell experiment, contact lenses were rinsed three times in sterile PBS (PBS-rinsed) or soaked in PBS for 24 h (PBS-soaked) under sterile conditions. Although lotrafilcon A lens packaging solution is based on phosphate buffer, lotrafilcon A was also submitted to similar PBS treatments for controls. Viability was measured by 3-(4,5-Dimethylthiazol-2-yl)-2,5-diphenyltetrazolium bromide (MTT) assay and expressed as a percentage relative to cells grown in the absence of a contact lens (control); n=3–5. The asterisk indicates significant difference from cells grown in the absence of a lens (p<0.005). The sharp symbol (hash mark) indicates significant difference from the PBS-rinsed lens, p<0.005.

To further examine the cytotoxic effect of the products released from lenses stored in borate-buffered packaging solution, two CH materials (etafilcon 1day and alphafilcon A) and two SH materials (galyfilcon A and balafilcon A) were rinsed or soaked for 24 h in PBS. As illustrated in Figure 3, soaking lenses in PBS for 24 h before cell exposure significantly increased cell viability, although a difference in the viability of control cells was still observed with galyfilcon A and balafilcon A (p<0.05). Simply rinsing contact lenses in PBS improved cell viability only for the borate-buffered lenses with galyfilcon A and alphafilcon A (p<0.035).

### Cell activation, integrin expression, and apoptosis

To characterize the phenotype of adherent cells at the end of the incubation with a contact lens, flow cytometry analysis was performed with a selection of materials (etafilcon A-1day, galyfilcon A, balafilcon A, and lotrafilcon A). Neither Fas, Fas ligand, nor ICAM-1 was upregulated in the presence of the contact lenses tested. In comparison, a significant decrease in the expression of α_3_ and β_1_ (Table 5) was observed with lenses stored in borate-buffered packaging solutions. Soaking the lenses in PBS for 24 h prevented any deleterious effect on integrin expression. A small decrease in β_4_ expression, although not statistically significant, was observed for cells incubated with lenses from borate-buffered packaging solution (Table 4). Adding balafilcon A borate-buffered packaging solution to the cell culture medium at a concentration of 10% also resulted in a significant reduction in α_3_, β_1_, and β_4_, in a magnitude similar to that observed from HCECs exposed to a balafilcon A lens (Table 6).

**Table 5:** Effect of packaging solution released from contact lens on integrin expression of corneal epithelial cells after incubation for 24 h.

**Integrin**	**Lens stored in borate buffer packaging solution**						**Lens stored in phosphate buffer packaging solution**	
	**etafilcon 1day**		**galyfilcon A**		**balafilcon A**		**lotrafilcon A**	
	**No treatment**	**PBS-soaked**	**No treatment**	**PBS-soaked**	**No treatment**	**PBS-soaked**	**No treatment**	**PBS-Soaked**
α_3_	72±5#	98±6	81±5*	100±4	72±8#	92±5	92±7	99±5
α_6_	93±9	95±4	95±14	94±5	95±11	100±9	93±4	96±6
β_1_	83±3*	95±5	85±6*	97±2	83±2*	98±3	98±3	100±0
β_4_	87±5	98±4	89±9	97±4	88±11	100±5	96±7	101±5

Cells were incubated for 24 h with contact lenses directly out of packaging or after a 24-h soak in PBS. Integrin expression is expressed as a percentage relative to its expression on cells grown in the absence of a contact lens (control). n=3 to 4, the asterisk indicates significantly different from control, p<0.05 and the sharp indicates significantly different from control, p<0.01.

**Table 6:** Integrin expression following a 24 h incubation with packaging solutions (final concentration: 10% diluted in medium) and borate buffer.

**Integrin**	**Balafilcon A packaging solution**	**Unisol 4 (BBS)**	**PBS**
α_3_	59±13*	75±3*	98±7
β_1_	71±7*	80±10*	98±3

Integrin expression is expressed as a percentage relative to cells grown in the absence of a contact lens (control). n=3, the asterisk indicates significantly different from cells grown in the absence of a lens p<0.05.

**Figure 4:**
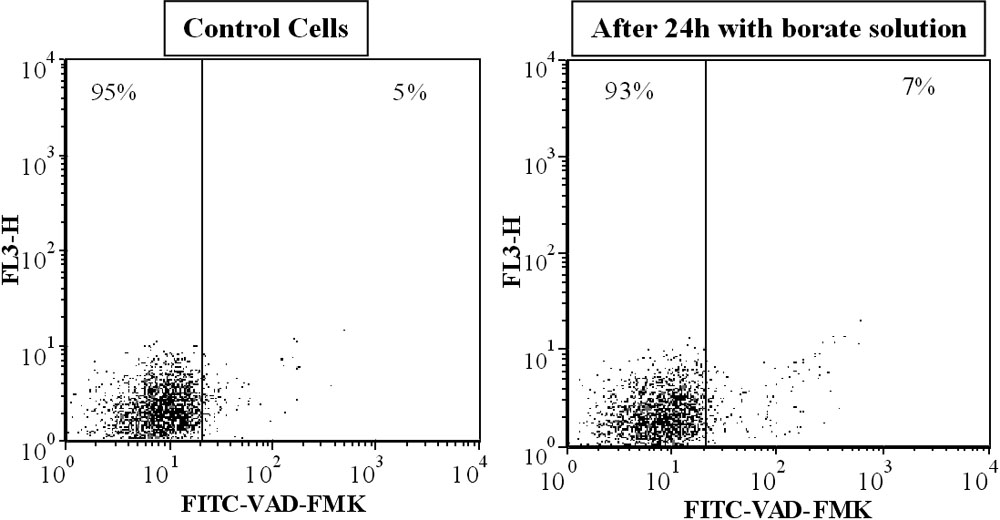
Caspase activation following incubation with phosphate or borate-buffered solution. No increase in cells staining positive for FITC-VAD-FMK was observed after cells were incubated for 24 h with either phosphate, borate-buffered solutions, or soaked lenses. Dot plots are representative of the three experiments that were performed.

**Figure 5:**
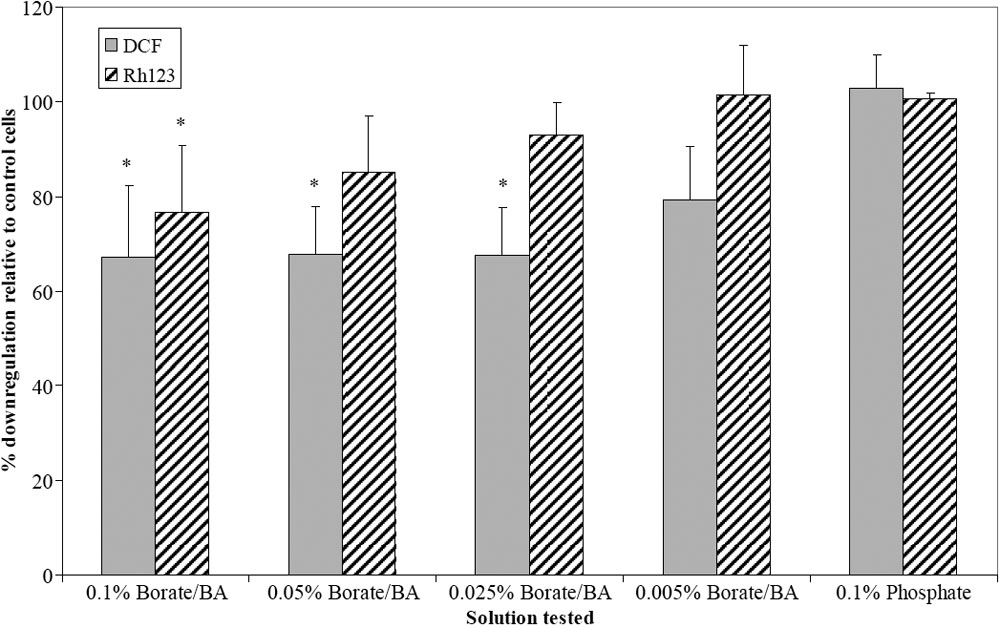
Effect of borate/boric acid on reactive oxygen species production and mitochondrial permeability. Cells were incubated for 24 h with different concentration of Unisol, a saline solution containing borate/boric acid. Reactive oxygen species (ROS) was measured with dichlorohydrofluorescein diacetate and mitochondrial permeability was assessed with Rh123. Results are expressed as a percentage relative to cells grown in the absence of a solution (control); n=3–4. The asterisk indicates significant difference from control, p<0.01.

As shown in Figure 4, caspase activation, detected using FITC-VAD-FMK, was not observed in cells incubated for 24 h with borate buffer-containing solutions. The percentage of cells staining positive for caspase activity (5±2%) was similar for cells grown in the absence of solution, or in the presence of PBS or Unisol. This was done with concentrations of up to 0.1% borate/boric acid (i.e., 20% Unisol), which were tested at 24 h; caspase activation remained within control levels. After 24 h exposure to diluted borate solutions, there was also no significant increase in binding of annexinV-FITC between cells exposed to diluted borate or phosphate solutions and control cells (cells incubated in the absence of solution and contact lens). For all samples, less than 2% of cells were found to bind Annexin V. In the presence of borate solution, a small but not significant increase in YO-PRO-1 permeability was observed (Table 7). To further characterize the potential mechanisms of borate-induced cytotoxicity, the intracellular levels of reactive oxygen species (ROS) were measured. As shown in Figure 5, a significant underproduction of DCF-A was observed for cells that had been exposed to borate-buffered solution for 24 h, either from products released from a lens or from direct dilution. A 25% reduction in fluorescence intensity of rhodamine 123 (Figure 5) was also observed with these samples. Upon phorbol myristate acetate (PMA) stimulation, all cells were able to increase their production of ROS (Table 8). As expected, the levels of PMA-induced ROS in cells exposed to diluted borate solutions for 24 h were lower than those of controls (since they had lower levels of ROS to start with). Only cells exposed to 0.1% borate/boric acid (20% unisol) for 24 h showed an impaired response in PMA-induced ROS formation. All other cells exhibited 50% increases in ROS production, suggesting that while they had an impaired level of intracellular ROS after 24 h-exposure to borate-buffered solution, they were still able to respond to PMA stimulus.

**Table 7:** YO-PRO 1 permeability on HCEC following 24 h exposure to phosphate and borate buffered solution.

**Medium alone**	**YO-PRO-1 expression (%)**	**cells staining positive for YOPRO-1 (%)**
Medium alone	100%	2.5±1.2
0.055% borate/boric acid*	121±20	3.0±1.7
0.025% borate/boric acid	105±7	2.6±0.8
0.0055% borate/boric acid	106±5	3.1±0.4
0.16% phosphate#	102±3	4.0±0.4

The asterisk corresponds to a 10% dilution of BBS (Unisol 4) in medium and the sharp corresponds to a 10% dilution of PBS in medium.

**Table 8:** PMA-induced ROS synthesis in HCEC after 24 h exposure to phosphate or borate buffered solutions.

**Sample**	**PMA-induced DCF Fluorescence intensity (absolute value)**	**Increase relative to unstimulated sample (%)**
Medium alone	95±8	152±8
0.10% borate/boric acid	44±6	126±14*
0.055% borate/boric acid	53±9	145±2
0.025% borate/boric acid	53±11	141±3
0.0055% borate/boric acid	71±11	161±2
0.16% phosphate	95±9	159±2

The asterisk indicates significantly different from control cells (medium alone), p<0.01.

## Discussion

While several in vitro studies have looked at the cytotoxicity of multi-purpose lens-cleaning solutions, there is little data available on the potential cytotoxicity of packaging solution on human corneal epithelial cells and how adsorbed compounds from the packaging solutions can be released from the contact lens and induce a cellular response in vitro. The results obtained from epithelial cells placed in contact with hydrogel contact lenses stored in phosphate-buffered packaging solution demonstrated almost 100% viability, which confirmed the validity of our in vitro onlay model, and also suggested that lenses stored in borate-buffered packaging solutions affect corneal epithelial cells in vitro. Because of its microbiological properties [27], borate buffer is used in many ophthalmic solutions, including contact lens disinfecting solutions and lubricating eye drops. The significant reduction in cell viability and the change in integrin expression in the presence of Unisol 4, an unpreserved borate-buffered solution, observed in our experiments further confirm the potential adverse effects of borate buffer on corneal epithelial cells in vitro, although their effect in vivo remains an area that requires further study.

While many researchers have studied the cytotoxicity of multi-purpose solutions, there are currently very few studies that have investigated borate buffer cytotoxicity, a buffer widely used in the ophthalmic industry. Borate buffer used in ophthalmic products is usually composed of boric acid (at less than 1%) and borate sodium (at less than 0.3%) in a 0.9% NaCl solution [28]. Poor in vivo corneal response to Unisol 4 has previously been reported by Chang et al. [29]. While Santodomingo-Rubido [18] looked at the cytotoxicity of boric acid on Chinese hamster’s lung fibroblasts, the potential cytotoxicity of borate buffer on HCECs does not appear to have been studied in vitro. Our study compared the borate buffer released from contact lenses to a 10% dilution of Unisol, which would be equivalent to a boric acid concentration of 0.055%. 1% boric acid has been shown to alter tight junctions on corneal epithelial cells [9]. Our results for the presence of Unisol 4 or borate-buffered packaging solutions, showing reduced viability and integrin expression on corneal epithelial cells, provide further information on the potential effect of boric acid at a lower concentration than that tested by Imayasu [9].

In vitro models using immortalized HCECs have been used recently to assess the potential cytotoxicity of ophthalmic solutions [7,9,11]. Contact lens extracts, dilution of solution, and short direct contact with ophthalmic solutions have all previously been tested on monolayer [8-11] and multilayer cell models [30]. However, to our knowledge, this is the first time that the potential cytotoxicity of solutions has been tested using an onlay model with corneal epithelial cells. Concurrent with our experiments, Maltseva et al. [20] reported the development of a similar in vitro model, by which cells at 20% confluence were grown in the presence of a contact lens for up to 3.5 days, to assess their response to a bacterial challenge. In agreement with our results, they did not observe any cytotoxic effect from the polymacon A lens, a lens stored in a phosphate-buffered packaging solution. Repeating their experiments, we found similar results for lenses stored in phosphate-buffered solution, but a significant reduction in cell proliferation (up to 50%) was observed for lens materials stored in borate-buffered packaging solution. While such an incubation model (20% confluency) is extreme for testing the potential release of cytotoxic products from a contact lens, it further emphasizes the deleterious effect that borate-buffered packaging solutions have on HCECs. This may have some implications for corneal wound healing. Further studies are warranted, to determine the specific mechanisms involved.

In the present study, both a 10% solution and the products directly released from contact lenses were examined. At 24 h of exposure, a difference in the results for viability and integrin expression was observed between direct tests of the packaging solution and tests of the solution released from contact lenses. This suggested that the products released from the contact lenses led to a final solution concentration equivalent to less than 10% of the borate buffer.

Due to their nature, there is always a concern that in vitro models may overestimate the cytotoxicity of a product. A monolayer of HCECs, as opposed to the multilayered structure of the cornea, was also used. Several experiments were performed to ensure that our results were not an artifact of the experimental conditions. Increasing cell culture time before stimulus or adding 10% serum did not change the observed cytotoxicity of lenses stored in borate-buffered packaging solution. Furthermore, in these experiments and others related to the solution released from contact lenses, we also observed that there was a difference between using a diluted solution and using a solution of products released directly from a lens. During various ongoing investigations in our laboratories, an increased cytotoxic response has generally been observed when using diluted solutions. This emphasizes the importance of testing products released from the materials, rather than testing the solution only, because the mechanisms and the profile of cytotoxicity may differ significantly, depending on both the chemistry of the lens and its surface treatment. Lens type has been previously observed to have a significant effect on the uptake and release of polyhexamethylene biguanide (PHMB)-containing solutions [31]. Our model was able to identify differences between lenses based on storage conditions. In vivo data on the effects of borate have shown a deleterious effect on rabbit corneas [29]. A recent clinical study [32] regarding the risk of microbial keratitis in daily disposable-lens wearers reported that lenses packaged in borate buffer (etafilcon A-1 day) were relatively less risky than lenses packaged in phosphate buffer (nelfilcon A). While the authors did not comment on packaging solutions, they discussed the more difficult handling of nelfilcon A, which could lead to corneal abrasions and possible increased predisposition to infection. Factors other than packaging solutions therefore appear to play a more important role in the risk for microbial keratitis.

In parallel to measuring the effect of packaging solutions on cell viability, integrin expression and markers of activation on adherent cells were also characterized. ICAM-1 is a receptor involved in leukocyte adhesion, which has been found to be upregulated upon cell activation [33,34]. The fact that ICAM-1 was not upregulated in the presence of the contact lens further confirms the material biocompatibility of contact lenses. In the presence of the release of borate-buffered packaging solution, reduced expression of α_3_, β_1_, and β_6_ was observed. Studies on corneal cell adhesion have shown integrin expression to be upregulated in the presence of specific extracellular matrix proteins or glycoproteins, such as laminin or collagen type IV [35]. Upon cell migration, which occurs as part of wound healing, integrin expression remains stable, although a relocation of the different integrins on the cell membrane occurs [36]. Thus, from our studies, the reduced integrin expression induced by the release of borate-buffered packaging solution suggests that the mechanism of adhesion of corneal epithelial cells has been adversely affected.

The α_3_ and β_1_ integrins have also been localized at cell-cell attachment junctions [37,38]. The observed reduction in α_3_ and β_1_ may also suggest that in our in vitro model, tight junctions were affected by the presence of 0.1% or less boric acid, and further supports observations for 1% boric acid made by Imayasu et al. [19]. α_3_ and β_1_ integrins have also been referred to as “survival integrins” [36], and their reduced expression in the presence of borate buffer may suggest that cells are undergoing apoptosis. As a means to further characterize the mechanisms involved in borate’s cytotoxicity to cells, various markers of apoptosis were assessed. Fas had been previously reported to be upregulated in conjunctival corneal cells following inflammatory stimulus [39,40]. The lack of Fas upregulation, the lack of staining activation for caspase, and the absence of significant changes in staining with YO-PRO-1 and Annexin V suggest that apoptosis is not being induced by borate-containing solutions, and that the stress from exposure to borate buffer-based solutions may lead to necrosis, rather than inducing apoptosis. It is also possible that a caspase-independent pathway such as endonuclease G, or a secondary necrosis, is being triggered, following exposure to borate/boric acid. Further investigations will be required to elucidate other potential apoptotic mechanisms. While the pathways of cell death induced by apoptosis have been well characterized, there is little known about mechanisms involved in necrosis [41]. The reduced level of DCF-A observed in cells following 24 h exposure to a low concentration of borate/boric acid may be indicative of previous extracellular release of ROS or impaired function. The lower mitochondrial permeability observed in cells after 24 h with borate-containing solution further suggest that the cells had been functionally damaged. Prior studies have shown both increased and decreased ROS production during short exposure to ophthalmic solutions [26,42]. After exposure to borate-containing solution for 2–4 h (data not shown), we observed an increase in ROS production (20–30%), which may explain the ROS underproduction and decreased mitochondrial permeability we observed at 24 h.

In conclusion, the in vitro contact lens onlay model reported in this study appears to be a valuable tool for studying the direct release of ophthalmic solutions on human corneal epithelial cells. These in vitro studies demonstrated that borate-buffered packaging solutions significantly affect corneal cell viability, as well as their adhesion phenotype. The potential cytotoxic effect of borate buffer on corneal epithelial cells was further demonstrated using a diluted solution of Unisol 4. Concentrations of borate/boric acid of less than 0.06% were shown to significantly alter cell phenotype. Our results suggest than necrosis, rather than apoptosis, is induced by exposure to borate-containing solutions, and that ROS may play a role. While borate-buffered saline is recognized for its microbiological properties, its relative cytotoxic effects on cells observed in vitro may outweigh the benefits of using it in packaging solutions, especially for daily disposable lenses, when corneas would be exposed to new, “soaked” lenses every day. Other parameters, such as packaging solution additives, mechanical factors, and regimen compliance, may also play a role. These need to be considered in clinical situations.

## References

[1] Efron N Morgan PB Cameron ID Brennan NA Goodwin M Oxygen permeability and water content of silicone hydrogel contact lens materials. Optom vis Sci 2007 84 328 37 17435503 10.1097/OPX.0b013e31804375ed

[2] Maldonado-Codina C Morgan PB In vitro water wettability of silicone hydrogel contact lenses determined using the sessile drop and captive bubble techniques. J Biomed Mater Res A 2007 83 496 502 17503532 10.1002/jbm.a.31260

[3] Carney FP Nash WL Sentell KB The adsorption of major tear film lipids in vitro to various silicone hydrogels over time. Invest Ophthalmol Vis Sci 2008 49 120 4 18172083 10.1167/iovs.07-0376

[4] Green-Church KB Nichols JJ Mass spectrometry-based proteomic analyses of contact lens deposition. Mol Vis 2008 14 291 7 18334948 PMC2254969

[5] Suwala M Glasier MA Subbaraman LN Jones L Quantity and conformation of lysozyme deposited on conventional and silicone hydrogel contact lens materials using an in vitro model. Eye Contact Lens 2007 33 138 43 17502748 10.1097/01.icl.0000244155.87409.f6

[6] Subbaraman LN Glasier MA Senchyna M Sheardown H Jones L Kinetics of in vitro lysozyme deposition on silicone hydrogel, PMMA, and FDA groups I, II, and IV contact lens materials. Curr Eye Res 2006 31 787 96 17050272 10.1080/02713680600888799

[7] Burgalassi S Chetoni P Monti D Saettone MF Cytotoxicity of potential ocular permeation enhancers evaluated on rabbit and human corneal epithelial cell lines. Toxicol Lett 2001 122 1 8 11397552 10.1016/s0378-4274(01)00261-2

[8] Dracopoulos A Dixon DG Jones LW Sivak JG Bantseev V In vitro assessment of medical device toxicity: interactions of benzalkonium chloride with silicone-containing and p-hema-containing hydrogel contact lens materials. Eye Contact Lens 2007 33 26 37 17224676 10.1097/01.icl.0000229775.17844.3f

[9] Imayasu M Shiraishi A Ohashi Y Shimada S Cavanagh HD Effects of multipurpose solutions on corneal epithelial tight junctions. Eye Contact Lens 2008 34 50 5 18180685 10.1097/ICL.0b013e318073cbdb

[10] Mowrey-McKee M Sills A Wright A Comparative cytotoxicity potential of soft contact lens care regimens. CLAO J 2002 28 160 4 12144238

[11] McCanna DJ Harrington KL Driot JY Ward KW Tchao R Use of a human corneal epithelial cell line for screening the safety of contact lens care solutions in vitro. Eye Contact Lens 2008 34 6 12 18180675 10.1097/ICL.0b013e31804fa141

[12] Chapman JM Cheeks L Green K Interactions of benzalkonium chloride with soft and hard contact lenses. Arch Ophthalmol 1990 108 244 6 2302109 10.1001/archopht.1990.01070040096038

[13] Schlitzer RL Preservative uptake by soft contact lenses. Contact Lens Spectrum 1992 7 41 3

[14] Rosenthal RA Dassanayake NL Schlitzer RL Schlech BA Meadows DL Stone RP Biocide uptake in contact lenses and loss of fungicidal activity during storage of contact lenses. Eye Contact Lens 2006 32 262 6 17099385 10.1097/ICL.0b013e31802b413f

[15] Lum E Perera I Ho A Osmolality and buffering agents in soft contact lens packaging solutions. Cont Lens Anterior Eye 2004 27 21 6 16303523 10.1016/j.clae.2003.11.002

[16] Cao J Jiang L Zhang X Yao X Geng C Xue X Zhong L Boric acid inhibits LPS-induced TNF-alpha formation through a thiol-dependent mechanism in THP-1 cells. J Trace Elem Med Biol 2008 22 189 95 18755394 10.1016/j.jtemb.2008.03.005

[17] Debbasch C Pisella PJ Rat P Warnet JM Baudouin C Cytotoxicity evaluation of three tear substitutes used in the treatment of dry eye syndromes. J Fr Ophtalmol 2000 23 863 9 11084443

[18] Santodomingo-Rubido J Mori O Kawaminami S Cytotoxicity and antimicrobial activity of six multipurpose soft contact lens disinfecting solutions. Ophthalmic Physiol Opt 2006 26 476 82 16918772 10.1111/j.1475-1313.2006.00393.x

[19] Griffith M Osborne R Munger R Xiong X Doillon CJ Laycock NL Hakim M Song Y Watsky MA Functional human corneal equivalents constructed from cell lines. Science 1999 286 2169 72 10591651 10.1126/science.286.5447.2169

[20] Maltseva IA Fleiszig SM Evans DJ Kerr S Sidhu SS McNamara NA Basbaum C Exposure of human corneal epithelial cells to contact lenses in vitro suppresses the upregulation of human beta-defensin-2 in response to antigens of Pseudomonas aeruginosa. Exp Eye Res 2007 85 142 53 17531223 10.1016/j.exer.2007.04.001

[21] Wu XY Cornell-Bell A Davies TA Simons ER Trinkaus-Randall V Expression of integrin and organization of F-actin in epithelial cells depends on the underlying surface. Invest Ophthalmol Vis Sci 1994 35 878 90 8125751

[22] Blazka ME, Diaco M, Harbell JW, Raabe H, Sizemore A, Wilt N, Bagley DM. Epiocular TM human cell construct: tissue viability and histological chnages following exposure to surfactants. Proceedings of the 5th World Congress on Alternatives and Animal Use in the Life Sciences. 2005.

[23] Papa V Leonardi A Getuli C Pacelli V Russo P Milazzo G Effect of ofloxacin and netilmicin on human corneal and conjunctival cells in vitro. J Ocul Pharmacol Ther 2003 19 535 45 14733711 10.1089/108076803322660459

[24] Caspase detection kit-QIA90-protocol. 2007.

[25] Dutot M Pouzaud F Larosche I Brignole-Baudouin F Warnet JM Rat P Fluoroquinolone eye drop-induced cytotoxicity: role of preservative in PX27 cell death receptor activation and apoptosis. Invest Ophthalmol Vis Sci 2006 47 2812 9 16799018 10.1167/iovs.06-0224

[26] Dutot M Warnet JM Baudouin C Rat P Cytotoxicity of contact lens multipurpose solutions: role of oxidative stress, mitochondrial activity and P2X7 cell death receptor activation. Eur J Pharm Sci 2008 33 138 45 18065213 10.1016/j.ejps.2007.10.006

[27] Houlsby RD Ghajar M Chavez GO Antimicrobial activity of borate-buffered solutions. Antimicrob Agents Chemother 1986 29 803 6 3729341 10.1128/aac.29.5.803 PMC284157

[28] Wanda R, Heiler DJ. Treatment of contact lenses with aqueous solutions including phosphonic compounds. 08/795116 [5858937], 1–21. 1999.

[29] Chang JH Ren HW Petroll MW Cavanagh DH Jester JV The application of in vivo confocal microscopy and tear LDH measurement in assessing corneal response to contact lens and contact lens solutions. Curr Eye Res 1999 19 171 81 10420187 10.1076/ceyr.19.2.171.5326

[30] Lim MJ Hurst RD Konynenbelt BJ Ubels JL Cytotoxicity testing of multipurpose contact lens solutions using monolayer and stratified cultures of human corneal epithelial cells. Eye Contact Lens 2009 35 287 96 19726996 10.1097/ICL.0b013e3181b9e92c

[31] Jayaraman S Intracellular determination of activated caspases (IDAC) by flow cytometry using a pancaspase inhibitor labeled with FITC. Cytometry A 2003 56 104 12 14608638 10.1002/cyto.a.10094

[32] Dart JKG Radford CF Minassian D Verma S Stapleton F Risk factors for microbial keratitis with contemporary contact lenses a case control study. Ophthalmology 2008 115 1647 54 18597850 10.1016/j.ophtha.2008.05.003

[33] Pereira HA Ruan X Gonzalez ML Tsyshevskaya-Hoover I Chodosh J Modulation of corneal epithelial cell functions by the neutrophil-derived inflammatory mediator CAP37. Invest Ophthalmol Vis Sci 2004 45 4284 92 15557434 10.1167/iovs.03-1052

[34] Pisella PJ Malet F Lejeune S Brignole F Debbasch C Bara J Rat P Colin J Baudouin C Ocular surface changes induced by contact lens wear. Cornea 2001 20 820 5 11685059 10.1097/00003226-200111000-00009

[35] Grushkin-Lerner LS Kewalramani R Trinkaus-Randall V Expression of integrin receptors on plasma membranes of primary corneal epithelial cells is matrix specific. Exp Eye Res 1997 64 323 34 9196383 10.1006/exer.1996.0207

[36] Stepp MA Corneal integrins and their functions. Exp Eye Res 2006 83 3 15 16580666 10.1016/j.exer.2006.01.010

[37] Kurpakus MA Daneshvar C Davenport J Kim A Human corneal epithelial cell adhesion to laminins. Curr Eye Res 1999 19 106 14 10420179 10.1076/ceyr.19.2.106.5330

[38] Takaoka M Nakamura T Ban Y Kinoshita S Phenotypic investigation of cell junction-related proteins in gelatinous drop-like corneal dystrophy. Invest Ophthalmol Vis Sci 2007 48 1095 101 17325151 10.1167/iovs.06-0740

[39] Brignole F De Saint-Jean M Goldschild M Becquet F Goguel A Baudouin C Expression of Fas-Fas ligand antigens and apoptotic marker APO2.7 by the human conjunctival epithelium. Positive correlation with class II HLA DR expression in inflammatory ocular surface disorders. Exp Eye Res 1998 67 687 97 9990333 10.1006/exer.1998.0566

[40] Brignole F Pisella PJ Goldschild M De Saint JM Goguel A Baudouin C Flow cytometric analysis of inflammatory markers in conjunctival epithelial cells of patients with dry eyes. Invest Ophthalmol Vis Sci 2000 41 1356 63 10798650

[41] Zong W-X Thompson CB Necrotic deah as a cell fate. Genes Dev 2006 20 1 15 16391229 10.1101/gad.1376506

[42] Huang C-C Cheng HH Cheng JS Tsai J-Y Liao W-C Fang YC Jan CR Tamoxifen-induced [Ca2+]i rise and apoptosis in corneal epithelial cells. Toxicology 2009 255 58 64 18992300 10.1016/j.tox.2008.10.001

